# Screening for *PRX* mutations in a large Chinese Charcot-Marie-Tooth disease cohort and literature review

**DOI:** 10.3389/fneur.2023.1148044

**Published:** 2023-07-04

**Authors:** Xinran Ma, Xiaoxuan Liu, Xiaohui Duan, Dongsheng Fan

**Affiliations:** ^1^Department of Neurology, Peking University Third Hospital, Beijing, China; ^2^Key Laboratory for Neuroscience, National Health Commission/Ministry of Education, Peking University, Beijing, China; ^3^Beijing Key Laboratory of Biomarker and Translational Research in Neurodegenerative Diseases, Beijing, China; ^4^Department of Neurology, China-Japan Friendship Hospital, Beijing, China

**Keywords:** Charcot-Marie-Tooth disease, periaxin, mutation, whole-exome sequencing, next-generation sequencing

## Abstract

**Background:**

Periaxins (encoded by *PRX*) play an important role in the stabilization of peripheral nerve myelin. Mutations in *PRX* can lead to Charcot-Marie-Tooth disease type 4F (CMT4F).

**Methods:**

In this study, we screened for *PRX* mutations using next-generation sequencing and whole-exome sequencing in a large Chinese CMT cohort consisting of 465 unrelated index patients and 650 healthy controls. Sanger sequencing was used for the validation of all identified variants. We also reviewed all previously reported *PRX*-related CMT cases and summarized the clinical manifestations and genetic features of *PRX*-related CMTs.

**Results:**

The hit rate for biallelic *PRX* variants in our cohort of Chinese CMT patients was 0.43% (2/465). One patient carried a previously unreported splice-site mutation (c.25_27 + 9del) compound heterozygous with a known nonsense variant. Compiling data on CMT4F cases and *PRX* variants from the medical literature confirmed that early-onset (95.2%), distal amyotrophy or weakness (94.0%), feet deformity (75.0%), sensory impairment or sensory ataxia (65.5%), delayed motor milestones (60.7%), and spinal deformity (59.5%) are typical features for CMT4F. Less frequent features were auditory impairments, respiratory symptoms, late onset, dysarthria or hoarseness, ophthalmic problems, and central nervous system involvement. The two cases with biallelic missense mutations have later onset age than those with nonsense or frameshift mutations. We did not note clear correlations between the type and site of mutations and clinical severity or distinct constellations of symptoms.

**Conclusion:**

Consistent with observations in other countries and ethnic groups, *PRX*-related CMT is rare in China. The clinical spectrum is wider than previously anticipated.

## 1. Introduction

Charcot-Marie-Tooth disease (CMT) is the most common form of inherited peripheral neuropathy (1) with a large degree of clinical and genetic heterogeneity. According to the results of electrophysiological studies especially the upper limb motor nerve conduction velocity (MNCV), CMT is classified into demyelinating forms, including CMT1 (autosomal dominant) and CMT4 (autosomal recessive); an axonal form, CMT2; and an intermediate form (2). Over 100 genes have been found to be associated with CMTs (https://neuromuscular.wustl.edu/).

CMT4F is a globally rare form of autosomal recessive inherited demyelinating CMT that is characterized by early-onset and slow progression (3). *PRX*, the causative gene for CMT4F (4, 5), was reported to be mutated in 1.2% (1/82) of a Chinese CMT cohort (6). *PRX* encodes two proteins, L-periaxin and S-periaxin, through alternative splicing (7). Periaxin is involved in the maintenance and stabilization of myelin (8). To date, over 60 pathogenic *PRX* mutations have been identified (Human Gene Mutation Database, Professional Version), most of which are nonsense and frameshift mutations (9). Splice-site mutations of *PRX* are rare. Due to the small number of reported CMT patients with *PRX* mutations, knowledge of PRX and CMT4F is still limited.

In this study, we screened for *PRX* mutations in a large Chinese CMT cohort and identified three additional CMT4F patients and a previously unreported splice-site mutation of *PRX*. We expanded the genetic and clinical spectrum of *PRX*-related CMT. We also reviewed previously reported CMT4F cases to provide a comprehensive overview of this rare form of CMT.

## 2. Methods

### 2.1. Materials and participants

Between January 2007 and February 2022, 465 unrelated index patients with CMT were enrolled in the CMT centers at Peking University Third Hospital and China-Japan Friendship Hospital. The family history, age of onset, clinical features, CMT neuropathy score, and electrophysiological features were collected in detail for each patient. Based on the electrophysiological criteria, 276 families were diagnosed with demyelinating CMT, 137 families were diagnosed with axonal CMT, and 52 families were diagnosed with intermediate CMT. Among the 465 index patients, 163 patients were diagnosed with CMT1A via multiplex ligation-dependent probe amplification (MLPA) and 88 patients underwent direct sequencing only for *PMP22, MPZ, GJB1*, and *MFN2*. The remaining 214 index patients and their relatives underwent whole-exome sequencing (WES) or next-generation panel testing screening. The present study was approved by the Ethics Committee of Peking University Third Hospital (IRB 00006761). Informed consent forms were signed by all the participants or their parents.

### 2.2. Genetic tests and bioinformatics analysis

#### 2.2.1. DNA extraction and gene sequencing

Peripheral blood samples were collected from all participants. Genomic DNA was extracted by a DNA isolation kit (Bioteke, AU1802). Concentration assays and quality control were performed using standard methods. Next-generation sequencing (NGS) targeting CMT-related genes including *PRX* (NM_181882.2) or WES was conducted in 214 index patients. All variants identified were then validated by Sanger sequencing for segregation analysis. For NGS, the sample preparation and sequencing procedure was performed following the Illumina instructions. The KAPA library preparation kit (Kapa Biosystems, KR0453) was used to prepare the DNA libraries. Pooled libraries were hybridized with capture probes on the Agilent SureSelect XT2 target enrichment system. Then sequencing targeting CMT-related genes was conducted on a HiSeq2500 platform. For WES, an Agilent Human All Exon V6 kit and HiSeq2500 platform were used. The direct Sanger sequencing was conducted on an ABI 3730XL DNA analyzer (Applied Biosystems, Waltham, MA, USA).

#### 2.2.2. Classification and assessment of mutations

The pathogenicity of the variants was assessed according to guidelines proposed by the American College of Medical Genetics and Genomics (ACMG) (10). Population databases, including dbSNP (http://www.ncbi.nlm.nih.gov/projects/SNP), 1000 Genomes (http://www.1000genomes.org/), GnomAD (http://gnomad.broadinstitute.org/), and Exome Sequencing Project (http://evs.gs.washington.edu/), and 650 healthy Chinese individuals were used as controls to exclude polymorphisms and benign variants. We used prediction software including Human Splicing Finder version 3.1 (http://www.umd.be/HSF/) for *in silico* analysis of variants.

### 2.3. Literature review

We reviewed all previously reported *PRX*-related CMT4F cases in PubMed. The Human Gene Mutation Database, Professional Version (HGMD pro), and ClinicalVar database were searched for all reported pathogenic mutations. Mutations identified as pathogenic and likely to be pathogenic were included, while those with unknown significance or with a diagnosis other than CMT were excluded.

## 3. Results

### 3.1. Clinical manifestation

In total, 465 unrelated patients diagnosed with CMT were enrolled in this study. Pathogenic mutations of *PRX* were detected in two patients, including one isolated case and another individual who had an affected sibling. Their detailed clinical data are shown in Table 1.

**Table 1:** Clinical features of the three CMT4F patients identified in this study.

**Patient**			**1**	**2**	**3**
Mode of inheritance			AR	AR	AR
Gender/age (year) at onset			F/1	M/1	F/1
Age at examination (year)			22	21	12
Disease duration (year)			21	20	11
Cranial nerve involvement			Auditory	Normal	Normal
Motor	Muscle atrophy	UL	N	N	Y
		LL	N	Y	Y
	Muscle strength^a^	Intrinsic hand muscles	V	V	V-
		Feet	V-	V-	IV-
Sensory	Pinprick		Below ankle	Below ankle	Below ankle
	Vibration		Below knee	Below ankle	Below ankle
Tendon reflexes			UL(–), LL(+)	NA	NA
Ulnar nerve CMAP amplitude (mV)			Absent	0.7	0.5
Ulnar nerve MNCV (m/s)			Absent	19.8	14.2
Ulnar nerve SNAP amplitude (μV)			Absent	Absent	Absent
Ulnar nerve SNCV (m/s)			Absent	Absent	Absent
CMTNS			18	14	16
Other clinical manifestations			Delayed motor milestones, pes cavus, sensory ataxia	Delayed motor milestones, sensory ataxia	Delayed motor milestones, sensory ataxia, foot surgery and pes cavus, scoliosis

^a^Medical Research Council Scale was used for the grading of muscle strength.

AR, autosomal recessive; N, no; Y, yes; F, female; M, male; UL, upper limbs; LL, lower limbs; NA, not available; CMAP, compound muscle action potential; MNCV, motor nerve conduction velocity; SNAP; sensory nerve action potential; SNCV, sensory nerve conduction velocity; CMTNS, Charcot–Marie–Tooth neuropathy score.

**Figure 1:**
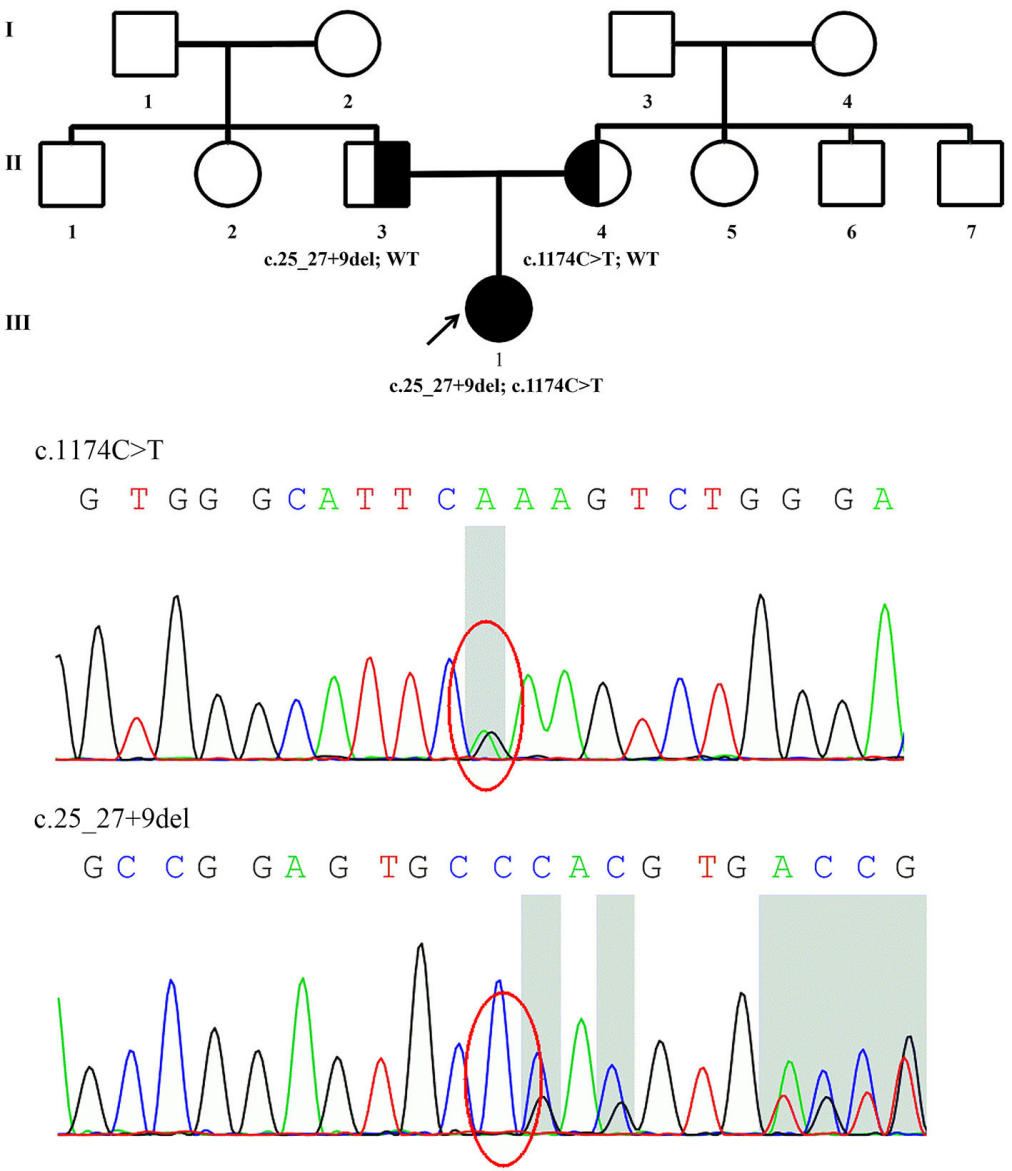
Family pedigree of Patient 1 and genomic sequencing electropherograms. A novel *PRX* splice-site mutation was detected. The reverse strand is shown for the c.1174C>T variant. WT, wild type.

Patient 1 was a 28-year-old woman who was the only child of healthy, non-consanguineous parents and had no affected relatives (Figure 1). Her mother took cold medicine during pregnancy. Patient 1 presented with delayed motor milestones and subsequently presented with motor deficits and unsteady gait. No intellectual disability or vision problems were reported. Her symptoms progressed slowly in the following 28 years. On her visit at the age of 22 years, no abnormality of the cranial nerve was found on physical examination. Severe sensory ataxia was observed. She also had pes cavus but no scoliosis. Pinprick sensitivity was decreased in the distal lower limbs. Vibration sensation was absent below the knees. Mild weakness was present in the feet only, with no severe muscle atrophy. Tendon reflexes were absent in the bilateral upper limbs and decreased in the lower limbs. No obvious abnormalities were found on the brain and whole-spine magnetic resonance imaging except for cervical intervertebral disk herniation. She underwent electrophysiological examinations at the age of 22 years, and the compound muscle action potential (CMAP) and sensory nerve action potential (SNAP) of the bilateral ulnar and median were undetectable in nerve conduction studies. The distal latency of CMAP was prolonged in the bilateral facial nerves, indicating demyelinating neuropathies. She also presented with prolonged latency in the waves III and V of the right side during an brainstem auditory evoked potential (BAEP) test. No biopsy was conducted. She complained of tinnitus starting at the age of 27 years. At age 28, the hearing test was conducted, and bilateral impairment of high-frequency sound was detected.

Patient 2 was a 21-year-old man who had a history of delayed motor milestones, with independent walking delayed until 3 years old, balance problems, and numbness starting at 16 years of age. He was the first child of healthy, non-consanguineous parents from the same region. He had a younger affected sister and three healthy siblings. Physical examination showed severe sensory ataxia and sensory loss in the distal lower limbs. Weakness was mild. Nerve conduction studies indicated severe demyelination and axonal neuropathy.

Patient 3 was a 12-year-old girl who was the younger sister of Patient 2. She had delayed motor milestones, with independent walking delayed until 26 months of age. During childhood, she developed gait difficulties and balance problems. The symptoms progressed moderately. On examination at age 12, she had scoliosis and bilateral pes cavus. She had moderate to severe weakness and atrophy in the distal part of her lower limbs. Her Romberg sign was positive. Vibration sensation was absent in her lower limbs. Electrophysiological studies showed demyelinating neuropathy with mildly reduced CMAP amplitude.

### 3.2. Genetic results

The details of all identified pathogenic or likely pathogenic mutations are shown in Table 2. Compound heterozygosity for *PRX* variants was detected in Patient 1. The mutation p.R392X is a previously reported pathogenic mutation (11), while c.25_27 + 9del is a novel splice-site mutation of *PRX*. This novel deletion mutation alters the exon 4-intron 4 junction. *In silico* analysis using Human Splicing Finder showed this mutation will inevitably destroy the intron four donor splice site. According to the ACMG guidelines, this novel mutation has very strong evidence for pathogenicity. Segregation studies showed that the mutations were located on different parental chromosomes (Figure 1). A homozygous mutation p.R953X (4) was found in the two siblings (Patients 2 and 3). This mutation was segregating in their family and their parents were heterozygous carriers.

**Table 2:** Genotypes of the three CMT4F patients.

**No**.	**Location**	**cDNA change**	**Protein change**	**Frequency in population database**				***In silico* analysis**	**References**	**ACMG**
				**1000 Genomes**	**GnomAD (global/Asian)**	**ESP**	**650 controls**			
1	Exon 7	c.1174C>T (rs773009397)	R392X	0	0.000024/0.000020	0	0	NA	(11)	Pathogenic
	Exon4-intron 4	c.25_27+9delGAGGTGAGTGCC	Splice error	0	0	0	0	Donor splice-site disrupted,	Novel	Pathogenic (PVS1, PM2-3, PP3-4)
2, 3	Exon 7	c.2857C>T (rs104894714), homozygous	R953X	0	0.000032/0.000040	0	0	NA	(4)	Pathogenic

NA, not applicable; PVS, very strong evidence of pathogenicity; PM, moderate evidence of pathogenicity; PP, supporting evidence of pathogenicity.

Refseq: NM_181882.2.

### 3.3. Literature review of *PRX-*related CMTs

**Figure 2:**
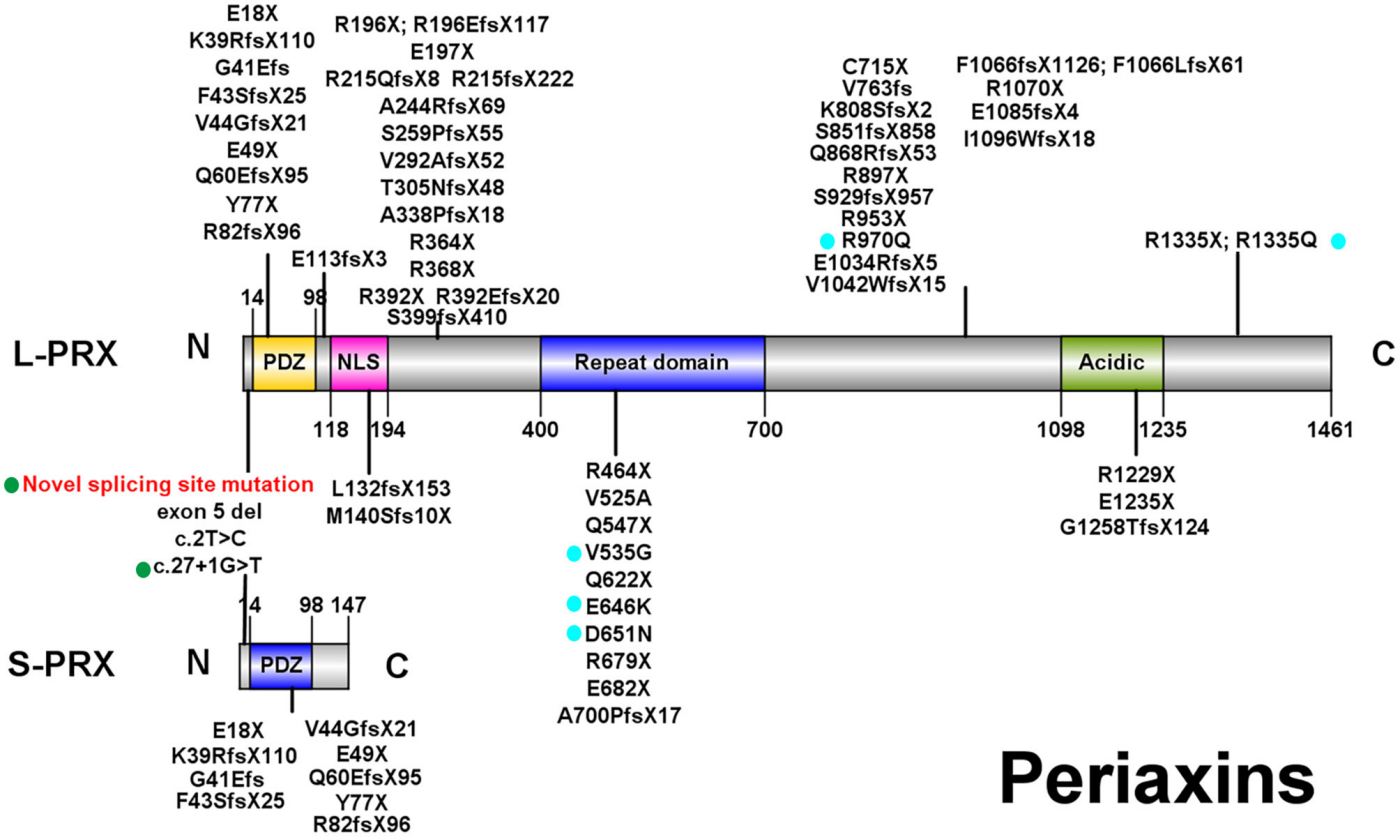
Illustrations of PRX. All pathogenic or likely pathogenic mutations of PRX from previously reported CMT4F cases and this study are shown. Green points: splice-site mutations. Blue points: nonsense and frameshift mutations. PDZ, PSD-95/Discs Large/ZO-1 domain; NLS, nuclear localization signal domain.

Currently, over 60 *PRX* mutations that are either pathogenic or likely pathogenic (Figure 2; those with a diagnosis other than autosomal recessive CMT were excluded) have been derived from nearly 70 CMT4F pedigrees (Supplementary Table).

Eighty-four patients have available clinical information, and their clinical features are summarized in Table 3. The symptoms usually start in the first decade of life, and only four patients (4.8%) have late onset (after 20 years old). The most frequent symptoms are distal weakness or muscle atrophy (94%), feet deformity (75%), sensory impairment or sensory ataxia (65.5%), delayed motor milestones (60.7%), and spinal deformity (59.5%). Auditory impairments, ophthalmic symptoms, and respiratory symptoms are reported in 16.7%, 16.7%, and 8.3% of patients, respectively. CNS involvement is recorded in 16 patients (19.0%), including epilepsy in eight patients (9.5%), structural abnormality in six patients (7.1%), intellectual disability in four patients (4.8%), and pyramidal and cerebellar signs in two and one patients, respectively. Dysarthria or hoarseness is recorded in three CMT4F patients. Symptoms occasionally recorded in CMT4F patients include diabetes (12), congenital loss of nails (13), tongue fasciculation (14, 15), asymmetrical involvement (16), proximal weakness (15), and trigeminal neuralgia (17).

**Table 3:** Clinical features of all previously reported CMT4F cases.

**Clinical feature**		**Number of patients**	**Frequency (%)**
Distal weakness or muscle atrophy		79	94.0
Feet deformity		63	75.0
Sensory impairments or sensory ataxia		55	65.5
Delayed motor milestones		51	60.7
Spinal deformity		50	59.5
Auditory impairments^a^		14	16.7
Respiratory symptoms		7	8.3
Late-onset (after 20 years of old)		4	4.8
Dysarthria or hoarseness		3	3.6
Ophthalmic symptoms	Total^a^	14	16.7
	Ophthalmoplegia^a^	9	10.7
	Glaucoma	2	2.4
	Myopia	1	1.2
	Cataract	1	1.2
	Maculopathy	1	1.2
CNS involvement	Total	16	19.0
	Epilepsy^a^	8	9.5
	Structural abnormality	6	7.1
	Intellectual disability	4	4.8
	Pyramidal signs	2	2.4
	Cerebellar signs	1	1.2

Eighty-four patients with detailed clinical information were included.

^a^Including inferred data because the exact number was unavailable.

Nerve conduction studies usually show a dramatic decrease in MNCV and undetectable SNAP (18). Elevated protein levels in cerebrospinal fluid have been reported (19). In one autopsy case, enlarged spinal nerve roots and demyelination of the phrenic, recurrent laryngeal, and oculomotor nerves were found (20, 21). Biopsy usually shows demyelination, classic onion bulbs, or sometimes the formation of basal lamina onion bulbs, tomacula, and myelin folding (18).

Currently, two cases with biallelic missense *PRX* mutations have been reported (22, 23). Their symptoms started at the age of 18 and 28, respectively, which appeared later than other patients. However, this needs more confirmation. No other obvious correlations between the type and site of mutations and clinical severity or distinct constellations of symptoms were found.

## 4. Discussion

We screened for *PRX* mutations in a large Chinese CMT cohort via NGS and WES. Two index patients with *PRX* mutations were found in the 465 CMT patient cohort. They were diagnosed with CMT4F. To the best of our knowledge, this is the largest Chinese CMT cohort to date and the first *PRX* screening study in China, which helps to clarify the distribution and characterization of *PRX*-related CMT in China. A splice-site mutation of *PRX* was identified, which is very rare in *PRX*-related CMT. Our study expanded the genetic spectrum of *PRX*-related CMT.

In our study, *PRX* mutations contributed to 0.43% of CMT cases (CMT1A included). In a previous study in the Chinese population, 1.2% (1/82) (6) of CMT cases were linked to *PRX* mutations. The discrepancy may be due to differing sample sizes, sequencing methods, or geographic distribution of patients. The frequency we reported is similar to previous studies from other countries, such as 0.4% in Japan (24), 1.6% in Spain (17), 0.35% in Canada (25) (2% of positive results), and 0.3% in a cross-country study (26).

*PRX*, located in chromosome 19 and containing seven exons (4), encodes two isoforms of periaxin through alternative splicing (7). L-periaxin (exon 4, 5, 6, and 7) and S-periaxin (exon 4, 5, and 6 and intron 6) share the same PSD-95/Discs Large/ZO-1 (PDZ) domain, which mediates the interaction between periaxin and other proteins (4, 7). L-periaxin also contains a nuclear localization signal (NLS) domain, a repeat domain, and an acidic domain (4) (Figure 2). L-periaxin links the cytoskeleton of Schwann cells to the basal lamina via its interactions with other proteins. The basic domains in the N-terminal of L-periaxin, which also function as NLS domains, bind with Drp2 and form the Prx/Drp2/Dag complex (27). The C-terminal of L-periaxin influences the function of this complex (28) and binds with β4 integrins (29). L-periaxin also binds with ezirin, which links the membrane and cytoskeleton, through both N- and C-terminals (30). The function of S-periaxin is not fully understood. Currently, it is believed to regulate the function of L-periaxin (31, 32). The loss of periaxin leads to instability of the peripheral nerve myelin and demyelinating neuropathy in murine models (8). Periaxin has recently been found to be expressed in the endothelium of small vessels in the human brain and influences the function of the blood–brain barrier (33).

Considering both the available literature and our study, the typical *PRX*-related CMT phenotype is characterized by early onset and slow progression of the disease, predominant sensory involvement, sensory ataxia, and distal amyotrophy or weakness (12, 18). Scoliosis is usually present in *PRX*-related CMT patients over the age of 12 years (12) but was only found in one of our patients.

As more cases have been reported, *PRX*-related CMT has shown a certain variability in clinical manifestations. A few patients may have late onset (22) or rapid onset (14). Newly recognized manifestations include auditory problems (12, 14, 15), ophthalmic problems (12), dysarthria (12, 23), hoarseness (22), vocal cord palsy (20), respiratory problems (12, 20), and involvement of the CNS (16, 23, 34–37). Abnormal BAEPs are common in CMT cases. The previously reported *PRX*-mutated CMT cases showed wave I delay (14, 15), which indicated auditory nerve lesions. In our study, Patient 1 presented with delayed waves III and V in BAEP, which is common in demyelinating CMT (38). Prolonged latencies in vestibular-evoked myogenic potentials were also reported in other CMT4F cases (39). These findings indicate that auditory and vestibular pathways may also be impaired in CMT4F. Periaxin is also expressed in lens fibers and is involved in the maintenance and development of the lens (40). Heterozygous mutations in *PRX* have been detected in pedigrees with congenital cataracts (41, 42). This may partially explain the ophthalmic symptoms, such as myopia and cataracts, observed in CMT4F. Cranial nerve demyelination was confirmed by pathology (20). The involvement of the phrenic and recurrent laryngeal nerves in CMT4F needs more attention because respiratory problems are sometimes fatal (20). Manifestations of CNS involvement include epilepsy (37, 43), delayed development of intelligence and cognitive deficits (36, 37), pyramidal signs (16), cerebellar signs (23), and structural abnormalities (34, 35, 37). In one case, this may be unrelated to the *PRX* mutation (37). The role of periaxin in the blood–brain barrier may partially explain these phenotypes (33). More research is needed to be carried out to clarify the function of periaxin in the CNS. However, the rare symptoms are not commonly observed and might be coincidental rather than part of the phenotype.

Consistent with a previous study (11), no obvious phenotype–genotype correlations were found in CMT4F in our study except that the onset age seems to be later in those with biallelic missense mutations. Over 60 pathogenic *PRX* mutations were detected. Most of them are nonsense or frameshift mutations, suggesting that a loss of function is the pathogenic mechanism for *PRX*-related neuropathies. Most mutations are located in exon 7, which is the largest exon encoding 90% of the protein (44). Currently, there are a few known pathogenic mutations for CMT which affect both L- and S-periaxin, including our novel splice-site mutation c.25_27 + 9del. Similar to our family with the splice-site variant, most patients with mutations affecting L- and S-periaxin did not develop symptoms distinct from those observed in patients with mutations in exon 7. However, a report on a single family with a homozygous p.Y77X mutation suggested that mutations affecting both types of periaxin could lead to a more severe phenotype (43).

## 5. Conclusion

We have elucidated the clinical and genetic features of *PRX*-related CMTs and reported a novel splice-site mutation, c.25_27 + 9del. *PRX*-related CMT accounted for 0.43% of the whole CMT cohort (CMT1A included) in China. The review of the literature showed that most CMT4F cases presented with early-onset and slow-progression demyelinating CMT. The discovery of rare symptoms indicates that the clinical spectrum is wider than previously anticipated.

## Data availability statement

The datasets presented in this study can be found in online repositories. The data presented in the study are deposited in the GenBank, accession number PRJNA946142. Further inquiries can be directed to the corresponding author.

## Ethics statement

The studies involving human participants were reviewed and approved by Ethics Committee of Peking University Third Hospital. Written informed consent to participate in this study was provided by the participants' legal guardian/next of kin. Written informed consent was obtained from the individual(s), and minor(s)' legal guardian/next of kin, for the publication of any potentially identifiable images or data included in this article.

## Author contributions

XL conceived the study, established the cohort, and revised the manuscript. XM analyzed the data and wrote the manuscript. XD and DF participated in the establishment of the cohort and provided technical support. All authors contributed to the article and approved the submitted version.
