# Supplementary material for: Screening for PRX mutations in a large Chinese Charcot-Marie-Tooth disease cohort and literature review
Source: Front Neurol. 2023 Jul 4;14:1148044. doi: 10.3389/fneur.2023.1148044 (PMC10352492; doi:10.3389/fneur.2023.1148044)
Supplement: Supplementary file 1 [file Table_1.docx]

Supplementary Material

Screening for *PRX* mutations in a large Chinese Charcot-Marie-Tooth disease cohort and literature review

Xinran Ma, Xiaoxuan Liu^*^, Xiaohui Duan, Dongsheng Fan

*** Correspondence:** Xiaoxuan Liu: lucyan_liu@bjmu.edu.cn

## Supplementary table. Summary of all previously reported *PRX*-related CMT cases.

| **Ref** | **Country/region** | **Mode of inheritance/no. of patients** | **Diagnosis** | **Onset age**  **(y)** | **Age at exam** | **Core symptoms** | **Additional symptoms** | **CMTNS** | **MCV**  **(m/s)** | **Nucleotide change** | **Protein change** |
| --- | --- | --- | --- | --- | --- | --- | --- | --- | --- | --- | --- |
| Cases from Northern America | | | | | | | | | | | |
| (1) | USA  Castoro R et al (2023) | Sporadic | CMT4F | 0 | 5 | Distal muscle amyotrophy and weakness, distal sensory impairment, pes cavus | Diffuse hypotonic since birth; scoliosis | NA | NA | c.2857C > T  (homozygous) | p.R953X |
| (2) | Canada  Volodarsky M et al. (2021) | NA | CMT | NA | NA | NA | NA | NA | NA | c.122_124delinsAAGCTGCGCGAGGAAGCTGC  (homozygous) | p.G41EfsX32 |
|  |  | NA | CMT | NA | NA | NA | NA | NA | NA | c.165_177dupGAGCCTCAGCCTG; c.2857C>T; c.2T>C; c.3198delT; c.3286_3356del;  c.4003C>T | p.Q60EfsX95;  p.R953X;  NA;  p.F1066LfsX61  p.I1096fsX18;  p.R1335X |
| Cases from Europe | | | | | | | | | | | |
| (3, 4) | Poland  Kabzinska D et al.  (2006, 2009) | Sporadic | CMT4F | 5 | 8 | Delayed motor mile stones, distal muscle weakness, sensory ataxia, sensory impairment, pes cavus, broad-based gait with steppage |  | NA | Absent | c.1194_1197delTTCC  (homozygous) | p.S399fsX410 |
|  |  | NA | NA | NA | NA | NA | NA | NA | NA | c.2909G>A;  NA | p.R970Q;  NA |
| (5) | Romania  Barankova et al. (2008) | Sporadic | CMT4F | <1 | 5 | Delayed motor milestones, pes cavus, unsteady gait, normal sensation and strength on examination | hypotonia | NA | 17 | c.3286_3356del71  (homozygous) | p.I1096fsX18 |
| (6) | Italy  Marchesi C et al.  (2010)  Citrigno L et al. (2020)  Crimi M et al. (2022) | AR/2 | CMT4F | <1  <1 | 43  45 | Delayed motor milestones, pes cavus, slow progression, distal sensory impairment, distal weakness and amyotrophy, steppage gait 2/2, ataxic gait 1/2, sensory ataxia 1/2 | Scoliosis 2/2 | 21  26 | 13.3  5.6 | c.1639C>T, c.2421delC | p.Q547X;  p.K808SfsX2 |
|  |  | AR/2 | CMT4F | <1 | 34 | Delayed motor milestones, pes cavus, ataxic gait, sensory ataxia, distal weakness, distal sensory impairment | Scoliosis | 18 | NA | c.2044G>T  (homozygous) | p.E682X |
|  |  | Sporadic | CMT4F | <1 | 38 | Delayed motor milestone, pes cavus, distal weakness and amyotrophy, steppssage and ataxic gait, sensory ataxia, distal sensory impairment | Scoliosis | 24 | 3 | c.773_774dupCC  (homozygous) | p.S259PfsX55 |
| (7) |  | NA | CMT4F | 36 | 66 | Slowly progressive mild phenotype, slight reduction in NCVs and prominent sensory involvement, ataxic gait, distal amyotrophy and weakness, distal sensory impairment | Late-onset | NA | 31 - 34 | c.3286_3356del71  (homozygous) | p.I1096fsX18 |
| (8) |  | Sporadic | CMT4F | 2 | 46 | Delayed motor milestone, distal weakness, steppage gait | Lateral asymmetry, hyperreflexia, | NA | NA | c. 1639C>T  (homozygous) | p.Q547X |
| (9, 10) | Belgium  Baets J et al. (2011)  Takashima et al.  (2002) | AR/2 | DSN | <1 | 50 | Delayed motor milestones, distal weakness and amyotrophy, sensory ataxia, distal sensory impairment | Hearing loss 2/2, scoliosis 2/2 | NA | 3.0 | c.2145T>A  (homozygous) | p.C715X |
| (11) | France  Renouil M et al.  (2013)  Hoebeke C et al.  (2018) | AR/6, sporadic/8 | CMT4F | Most in infancy | 46,  55,  52,  53,  13,  14,  43,  51,  31,  12,  11 | Delayed acquisition of walking, foot deformity, distal weakness and amyotrophy | Deafness 1/14, glaucoma 2/14, dysarthria 1/14, strabismus 1/14, myopia 1/14, diabetes 1/14, maculopathy 1/14, respiratory symptoms 5/14, scoliosis 7/14, | 21,  29,  34,  19,  16,  16,  17,  17,  22,  14 | Average  10 m/s | c.3253dupG  (homozygous) | p.E1085fsX4 |
|  |  | AR/2 | CMT4F | 2,  2 | 52,  49 | Delayed acquisition of walking, pes cavus, distal weakness and amyotrophy | Scoliosis 1/2 | 18,  NA |  | c.3198delT  (homozygous) | p.F1066fsX1126 |
|  |  | AR/2 | CMT4F | 2,  2 | 43,  41 | Delayed acquisition of walking, distal weakness and amyotrophy | Strabismus 1/2, scoliosis 2/2, respiratory symptoms 1/2 | 35,  35 |  | c.642insC; c.2553_2556delTTC | p.R215fsX222; p.S851fsX858 |
|  |  | Sporadic | CMT4F | 2.5 | 29 | Pes cavus, distal weakness and amyotrophy | Respiratory symptoms,  scoliosis | 34 |  | c.116_117delAA; c.586C >T | p.K39RfsX110; p.R196X |
|  |  | Sporadic | CMT4F | 2 | 31 | Pes cavus, distal weakness and amyotrophy | Scoliosis | 35 |  | c.145G > T; c.1090C >T | p.E49X;  p.R364X |
| (12) |  | NA | NA | NA | NA | NA | NA | NA | NA | c.131_134del TTCG;  c.2689 C>T | p.V44GfsX21; p.R897X |
| (13) | Spain  Sivera R et al.  (2013) | AR/3 | CMT4F | 2.7 | 25.7 | Sensory ataxia, feet deformity, distal weakness, distal sensory impairment | Scoliosis 3/3,  trigeminal neuralgia 1/3 | 22.7 | 4.9 | c.589G>T; c.642insC | p.E197X;  p.R215QfsX8 |
|  |  | NA | CMT4F | 1 | 12 | Sensory ataxia, feet deformity, distal weakness, distal sensory impairment | Scoliosis | 18 | 5.8 | NA | p.E113fsX3 |
| (14) | Germany  Dohrn MF et al.  （2017） | AR | NA | NA | NA | NA | NA | NA | NA | c.1012delG  (homozygous) | p.A338PfsX18 |
|  |  | AR | NA | NA | NA | NA | NA | NA | NA | c.1390 C>T  (homozygous) | p.R464X |
|  |  | Sporadic | NA | NA | NA | NA | NA | NA | NA | c.913dupA; c.2602delC | p.T305NfsX48; p.Q868RfsX53 |
|  |  | Sporadic | NA | NA | NA | NA | NA | NA | NA | c.3120delA;  exon 5 deletion | p.V1042WfsX15;NA |
| Cases from Asia | | | | | | | | | | | |
| (15) | China  Sun B et al. (2017)  Yang Y et al. (2022)  Chen YH et al. (2020)  **This study** | NA | CMT4F | NA | NA | NA | NA | NA | NA | c.1390C>T  (homozygous) | p.R464X |
| (16) |  | Sporadic | CMT4F | NA | NA | NA |  | NA | NA | c.52G>T;  c.1390C>T | p.E18X;  p.R464X |
| (17) |  | Sporadic | CMT4F | 1.5 | 14 | Delayed motor milestone, ataxic gait, pes cavus, distal weakness and amyotrophy,distal sensory impairment, sensory ataxia | Scoliosis, CSF protein level elevated | NA | Absent | c.1174C>T  (homozygous) | p.R392X; |
|  |  | Sporadic | CMT4F | <1 | 22 | Delayed motor milestone, sensory ataxia, distal weakness, sensory impairment, pes cavus | Tinnitus, hearing loss | 13 | Absent | c.1174C>T;  c.25_27+9delGAGGTGAGTGCC | p.R392X;  splice-3 |
|  |  | AR/2 | CMT4F | 16  6 | 21  12 | Delayed motor milestones, sensory ataxia, ataxic gait, distal weakness and sensory impairment, pes cavus | Scoliosis 1/2 | 16  20 | 19.8  14.2 | c.2857C>T  (homozygous) | p.R953X |
| (18) | Japan  Kijima  Et al.  (2004)  Shimohata M et al.  (2005)  Otagiri et al.  (2006)  Abe A et al.  (2011)  Tokunaga et al.  (2012) | AR | CMT4F | <1 | 18 | Delayed motor milestone, distal weakness, normal sensation | mild increase in CSF protein level | NA | NA | c.3208C>T  (homozygous) | p.R1070X |
|  |  | AR | CMT4F | Childhood | 51 | Gait problem since childhood, distal weakness, pes cavus, distal sensory impairment | NA | NA | 20 | c.3208C>T  (homozygous) | p.R1070X |
|  |  | Sporadic | CMT4F | Childhood | 60 | Gait problem since childhood, distal weakness and amyotrophy, pes cavus, sensory impairment | NA | NA | 8 | c.3208C>T  (homozygous) | p.R1070X |
| (19) |  | AR | CMT4F | Childhood | 51 | Gait problem, distal muscle weakness,pes cavus, sensory impairment |  | NA | NA | c.3208C>T  (homozygous) | p.R1070X |
| (20) |  | Sporadic | DSN | <1 | 26 | Delayed motor milestones, distal muscle weakness and sensory impairment, ataxic gait | Mild elevated CSF protein level | NA | 2 | c.3208C>T; c.385_395insATCCAGAGTC | p.R1070X; p.L132FsX153 |
| (21) |  | AR (2)  Sporadic (3) | DSN  CMT4F | <10 |  | Demyelinating CMT | NA | NA | NA | c.875_906del32; c.915_923dup9; c.924A>T; c.927T>G  (homozygous) | p.V292AfsX52 |
| (22) |  | Sporadic | CMT4F | 28 | 65 | distal muscle weakness and amyotrophy, sensory impairment, delayed motor milestones (probably due to infantile paralysis) | Late onset, hoarseness | NA | Absent | c.1951G>A  (homozygous) | p.D651N |
|  |  | Sporadic | CMT4F | 50 | 63 | Distal muscle weakness, sensory impairment, pes cavus | Late onset | NA | 20.5 | c.3208C>T  (homozygous) | p.R1070X |
|  |  | Sporadic | CMT4F | 37 | 47 | Severe lower motor impairment, no pes cavus, normal sensation | Late onset, scoliosis | NA | 20.8 | c.3208C>T  (homozygous) | p.R1070X |
| (23) | Korea  Choi YJ et al. (2015) | Sporadic | DSN | <1 | 10 | Delayed motor milestones, ataxic gait, pes cavus, sensory impairment, sensory ataxia, distal weakness | Scoliosis | 20 | 2.6-3.0 | c.1174C>T;  c.2035C>T | p.R392X  p.R679X |
| (24, 25) | Lebanon  Guibot A et al.  (2001);  Delague V et al. (2000)  Megarbane A et al.  (2022) | AR/8 | CMT4F | <1 | NA | Delayed motor milestone, ataxic and steppage gait, pes cavus, distal weakness and amyotrophy, sensory ataxia, distal sensory impairment | Scoliosis 8/8, 2 of the patients have large cisterna magna | NA | Absent | c. 586C>T  (homozygous) | p.R196X |
| (26) |  | AR/10 patients in 3 families | CMT4F | 0-19(average 6) | NA | NA | NA | NA | NA | c. 586C>T  (homozygous) | p.R196X |
| (9) | Israel  Baets J et al. (2011)  Kuperberg M et al.  (2016) | Sporadic | DSN | Congenital | 11 | delayed motor milestone, pes cavus, ataxic gait | Moderate proximal  weakness, 47XXX  karyotype, hypotonia, | NA | NA | c.3772_3773delGGinsA  (homozygous) | p.G1258TfsX124 |
| (27) |  | AR | NA | NA | NA | Muscle weakness, hypotonia | Cataract, enlargement of temporal horns, bilateral hippocampal hypoplasia | NA | NA | c.418_419insG (homozygous) | p.M140Sfs10X |
| (28) | Israel and Palestine  Hengel H et al. (2020) | AR/2 | NA | NA | NA | NA | NA | NA | NA | c.27+1G>T  (homozygous) | NA |
| (29) | Turkey  Parman Y et al. (2004)  Auer-Grumbach M et al. (2008)  Yalcintepe S et al. (2021) | Sporadic | DSN | <2 | 4 | Delayed motor milestones, ataxic gait, distal weakness, sensory ataxia, pes cavus | Scoliosis | NA | 3 | c.3208C>T  (homozygous) | p.R1070X |
| (30) |  | AR/2 | CMT4F | 1-2 | NA | Delayed motor milestone, distal weakness, sensory ataxia | Kyphosis 1/2,  Congenital loss of nails | NA | Absent | c.2098delG  (homozygous) | p.A700PfsX17 |
| (31) |  | NA | NA | NA | NA | Gait disorder |  |  |  | c.3208C>T  (homozygous) | p.R1070X |
| (32) | Iran  Karakaya M et al, (2018) | AR/2 | CMT4F | 2 | NA | Distal weakness | Delayed cognition | NA | NA | c.3703G>T  (homozygous) | p.E1235X |
| (33) | Saudi Arab  Monies D et al. (2019) | AR/NA | CMT | NA | NA | NA | NA | NA | NA | c.586delC  (homozygous) | p.R196EfsX117 |
| (34) | Pakistan  Zaman Q et al.  (2023) | AR/8 | CMT4F | NA | NA | Pes cavus, steppage gait, sensory ataxia, amyotrophy and weakness | Bone deformities of feet and hands, bilateral scapular dyskinesis and winged scapula,  epilepsy in 6/8,  ptosis and blepharoptosis, scoliosis, hearing loss, tremor | NA | NA | c.231C>A  (homozygous) | p.Y77X |
| Cases from Africa | | | | | | | | | | | |
| (9) | Morocco  Baets J et al. (2011) | Sporadic | DSN | <1 | 4 | Delayed motor milestones |  | NA | 6.9 | c.730delG  (homozygous) | p. A244RfsX69 |
| (9, 10) | Maghreb  Baets J et al. (2011)  Takashima et al.  (2002) | Sporadic | DSN | <1 | 6 | Delayed motor milestones, amyotrophy, sensory ataxia, ataxic gait, distal weakness and amyotrophy | Slight delay of wave I on BAEP, scoliosis, tongue fasciculation | NA | NA | c.247delC  (homozygous) | p.R82fsX96 |
| (35) | Algeria  Nouioua et al.  (2011) | AR/4 | CMT4F | 12  10  10  7 | 22  20  17  15 | Ataxic gait, sensory ataxia, distal weakness and amyotrophy, pes cavus, distal sensory impairment | Scoliosis 4/4, late onset, pyramidal signs and hearing loss in 1 patient with bilateral acoustic neurofibroma and multiple cerebral meningiomas | NA | 9  8  5  6 | c.1090C>T  (homozygous) | p. R364X |
| (36) | Egypt  Makrythanasis P et al (2014) | AR/2 | NA | NA | 6  5 |  | Intellectual disability, developmental delay, hyperactivity, hypotonia, specific facies, epilepsy, hypogenesis of posterior part of corpus callosum | NA | NA | c.3099del  (homozygous) | p.E1034RfsX5 |
| Cases from uncertain area | | | | | | | | | | | |
| (37) | DiVincenzo C et  al. (2014) | NA | NA | NA | NA | NA | NA | NA | NA | c.124_125dup;  c.231C>G;  c.1173del;  c.1864C>T;  c.2689C>T;  c.3685C>T;  c.4003C>T | p.F43SfsX25; p.Y77X;  p.R392EfsX20; p.Q622X; p.R897X;  p.R1229X;  p.R1335X |
| (38) | SchabhuttlMet al. (2014) | Sporadic | HMSN | 18 | 57 | Muscle involvement of limbs, sensory impairment | Dysarthria, cerebellar signs, hypermobility of joints | NA | 24.8 | c.1574T>C;  c.4004G>A | p.V525A;  p.R1335Q |
| (39) | Boerkoel CF et al.  (2001) | AR/2 | DSN | <7 | 46 | Distal prominent motor involvement, no sensory ataxia, severe sensory impairment, pes cavus |  | NA | 3 | c.2857C>T; c.2787delC | p.R953X;  p.S929fsX957 |
|  |  | AR/1 | DSN | 1.5 | 6 | Delayed milestones, distal motor involvement, sensory impairment, sensory ataxia |  | NA | Absent | c.1102C>T;  c.2289delT | p.R368X;  p.V763fsX774 |
|  |  | AR/1 | DSN | 1 | 31 | Delayed milestones, distal motor involvement, sensory impairment, sensory ataxia, dysesthesia, pes cavus |  | NA | 2.1 | c.2787delC  (homozygous) | p.S929fsX957 |

Abbreviations: CMT: Charcot-Marie-Tooth disease; DSN: Dejerine-Sottas neuropathy; AR: autosomal recessive; MNCV: motor nerve conduction velocity; NCV: nerve conduction velocity; NA: not available; BAEP: brainstem auditory evoked potential; CSF: cerebrospinal fluid; CMTNS: Charcot–Marie–Tooth neuropathy score.

## Reference

1. Castoro R, Caress JB, Li J, Cartwright MS. Arg953* mutation in Periaxin causes CMT4F without nerve hypertrophy on ultrasound imaging: A case report and review of the literature. Clin Neurophysiol. 2023;147:14-6.doi:10.1016/j.clinph.2022.12.007

2. Volodarsky M, Kerkhof J, Stuart A, Levy M, Brady LI, Tarnopolsky M, et al. Comprehensive genetic sequence and copy number analysis for Charcot-Marie-Tooth disease in a Canadian cohort of 2517 patients. J Med Genet. 2021;58(4):284-8.doi:10.1136/jmedgenet-2019-106641

3. Kabzinska D, Drac H, Sherman DL, Kostera-Pruszczyk A, Brophy PJ, Kochanski A, et al. Charcot-Marie-Tooth type 4F disease caused by S399fsx410 mutation in the PRX gene. Neurology. 2006;66(5):745-7.doi:10.1212/01.wnl.0000201269.46071.35

4. Kabzinska D, Franaszczyk M, Kochanski A. [Charcot-Marie-Tooth disorders with autosomal recessive inheritance. Search for the molecular diagnostics model]. Med Wieku Rozwoj. 2009;13(2):146-53.

5. Barankova L, Siskova D, Huhne K, Vyhnalkova E, Sakmaryova I, Bojar M, et al. A 71-nucleotide deletion in the periaxin gene in a Romani patient with early-onset slowly progressive demyelinating CMT. Eur J Neurol. 2008;15(6):548-51.doi:10.1111/j.1468-1331.2008.02104.x

6. Marchesi C, Milani M, Morbin M, Cesani M, Lauria G, Scaioli V, et al. Four novel cases of periaxin-related neuropathy and review of the literature. Neurology. 2010;75(20):1830-8.doi:10.1212/WNL.0b013e3181fd6314

7. Citrigno L, Zoccolella S, Lastella P, Simone IL, Muglia M. A 71-nucleotide deletion in the periaxin gene in an Italian patient with late-onset slowly progressive demyelinating Charcot-Marie-Tooth disease. Eur J Neurol. 2020;27(10):2109-10.doi:10.1111/ene.14362

8. Crimi M, Tarawneh A. The genetic counseling in a patient affected by congenital polyneuropathy after a "diagnostic odyssey" recently solved with WES approach. Eur J Transl Myol. 2022;32(1).doi:10.4081/ejtm.2022.10361

9. Baets J, Deconinck T, De Vriendt E, Zimon M, Yperzeele L, Van Hoorenbeeck K, et al. Genetic spectrum of hereditary neuropathies with onset in the first year of life. Brain. 2011;134(Pt 9):2664-76.doi:10.1093/brain/awr184

10. Takashima H, Boerkoel CF, De Jonghe P, Ceuterick C, Martin JJ, Voit T, et al. Periaxin mutations cause a broad spectrum of demyelinating neuropathies. Ann Neurol. 2002;51(6):709-15.doi:10.1002/ana.10213

11. Renouil M, Stojkovic T, Jacquemont ML, Lauret K, Boue P, Fourmaintraux A, et al. [Charcot-Marie-Tooth disease associated with periaxin mutations (CMT4F): Clinical, electrophysiological and genetic analysis of 24 patients]. Rev Neurol (Paris). 2013;169(8-9):603-12.doi:10.1016/j.neurol.2013.07.004

12. Hoebeke C, Bonello-Palot N, Audic F, Boulay C, Tufod D, Attarian S, et al. Retrospective study of 75 children with peripheral inherited neuropathy: Genotype-phenotype correlations. Arch Pediatr. 2018;25(8):452-8.doi:10.1016/j.arcped.2018.09.006

13. Sivera R, Sevilla T, Vilchez JJ, Martinez-Rubio D, Chumillas MJ, Vazquez JF, et al. Charcot-Marie-Tooth disease: genetic and clinical spectrum in a Spanish clinical series. Neurology. 2013;81(18):1617-25.doi:10.1212/WNL.0b013e3182a9f56a

14. Dohrn MF, Glockle N, Mulahasanovic L, Heller C, Mohr J, Bauer C, et al. Frequent genes in rare diseases: panel-based next generation sequencing to disclose causal mutations in hereditary neuropathies. J Neurochem. 2017;143(5):507-22.doi:10.1111/jnc.14217

15. Sun B, Chen Z, Ling L, Yang F, Huang X. Clinical and genetic spectra of Charcot-Marie-Tooth disease in Chinese Han patients. J Peripher Nerv Syst. 2017;22(1):13-8.doi:10.1111/jns.12195

16. Yang Y, Ye S, Lyu Y, Xin H, Gao M, Ma J, et al. [Analysis of PRX gene variants in a child with Charcot-Marie-Tooth disease type 4F]. Zhonghua Yi Xue Yi Chuan Xue Za Zhi. 2022;39(7):749-53.doi:10.3760/cma.j.cn511374-20210311-00221

17. Chen YH, Zhang H, Meng LB, Tang XY, Gong T, Yin J. Novel mutation in the periaxin gene causal to Charcot-Marie-Tooth disease type 4F. J Int Med Res. 2020;48(2):300060519862064.doi:10.1177/0300060519862064

18. Kijima K, Numakura C, Shirahata E, Sawaishi Y, Shimohata M, Igarashi S, et al. Periaxin mutation causes early-onset but slow-progressive Charcot-Marie-Tooth disease. J Hum Genet. 2004;49(7):376-9.doi:10.1007/s10038-004-0162-3

19. Shimohata M, Hirahara K, Igarashi S, Hara K, Kijima K, Onodera O, et al. [A pedigree of Charcot-Marie-Tooth disease type 4F (Periaxin mutation)]. Rinsho Shinkeigaku. 2005;45(3):221-5.

20. Otagiri T, Sugai K, Kijima K, Arai H, Sawaishi Y, Shimohata M, et al. Periaxin mutation in Japanese patients with Charcot-Marie-Tooth disease. J Hum Genet. 2006;51(7):625-8.doi:10.1007/s10038-006-0408-3

21. Abe A, Numakura C, Kijima K, Hayashi M, Hashimoto T, Hayasaka K. Molecular diagnosis and clinical onset of Charcot-Marie-Tooth disease in Japan. J Hum Genet. 2011;56(5):364-8.doi:10.1038/jhg.2011.20

22. Tokunaga S, Hashiguchi A, Yoshimura A, Maeda K, Suzuki T, Haruki H, et al. Late-onset Charcot-Marie-Tooth disease 4F caused by periaxin gene mutation. Neurogenetics. 2012;13(4):359-65.doi:10.1007/s10048-012-0338-5

23. Choi YJ, Hyun YS, Nam SH, Koo H, Hong YB, Chung KW, et al. Novel Compound Heterozygous Nonsense PRX Mutations in a Korean Dejerine-Sottas Neuropathy Family. J Clin Neurol. 2015;11(1):92-6.doi:10.3988/jcn.2015.11.1.92

24. Guilbot A, Williams A, Ravise N, Verny C, Brice A, Sherman DL, et al. A mutation in periaxin is responsible for CMT4F, an autosomal recessive form of Charcot-Marie-Tooth disease. Human molecular genetics. 2001;10(4):415-21. doi: 10.1093/hmg/10.4.415

25. Delague V, Bareil C, Tuffery S, Bouvagnet P, Chouery E, Koussa S, et al. Mapping of a new locus for autosomal recessive demyelinating Charcot-Marie-Tooth disease to 19q13.1-13.3 in a large consanguineous Lebanese family: exclusion of MAG as a candidate gene. Am J Hum Genet. 2000;67(1):236-43.doi:10.1086/302980

26. Megarbane A, Bizzari S, Deepthi A, Sabbagh S, Mansour H, Chouery E, et al. A 20-year Clinical and Genetic Neuromuscular Cohort Analysis in Lebanon: An International Effort. J Neuromuscul Dis. 2022;9(1):193-210.doi:10.3233/JND-210652

27. Kuperberg M, Lev D, Blumkin L, Zerem A, Ginsberg M, Linder I, et al. Utility of Whole Exome Sequencing for Genetic Diagnosis of Previously Undiagnosed Pediatric Neurology Patients. J Child Neurol. 2016;31(14):1534-9.doi:10.1177/0883073816664836

28. Hengel H, Buchert R, Sturm M, Haack TB, Schelling Y, Mahajnah M, et al. First-line exome sequencing in Palestinian and Israeli Arabs with neurological disorders is efficient and facilitates disease gene discovery. Eur J Hum Genet. 2020;28(8):1034-43.doi:10.1038/s41431-020-0609-9

29. Parman Y, Battaloglu E, Baris I, Bilir B, Poyraz M, Bissar-Tadmouri N, et al. Clinicopathological and genetic study of early-onset demyelinating neuropathy. Brain. 2004;127(Pt 11):2540-50.doi:10.1093/brain/awh275

30. Auer-Grumbach M, Fischer C, Papic L, John E, Plecko B, Bittner RE, et al. Two novel mutations in the GDAP1 and PRX genes in early onset Charcot-Marie-Tooth syndrome. Neuropediatrics. 2008;39(1):33-8.doi:10.1055/s-2008-1077085

31. Yalcintepe S, Gurkan H, Dogan IG, Demir S, Sag SO, Kabayegit ZM, et al. The Importance of Multiple Gene Analysis for Diagnosis and Differential Diagnosis in Charcot Marie Tooth Disease. Turk Neurosurg. 2021;31(6):888-95.doi:10.5137/1019-5149.JTN.33661-21.3

32. Karakaya M, Storbeck M, Strathmann EA, Delle Vedove A, Holker I, Altmueller J, et al. Targeted sequencing with expanded gene profile enables high diagnostic yield in non-5q-spinal muscular atrophies. Hum Mutat. 2018;39(9):1284-98.doi:10.1002/humu.23560

33. Monies D, Abouelhoda M, Assoum M, Moghrabi N, Rafiullah R, Almontashiri N, et al. Lessons Learned from Large-Scale, First-Tier Clinical Exome Sequencing in a Highly Consanguineous Population. Am J Hum Genet. 2019;104(6):1182-201.doi:10.1016/j.ajhg.2019.04.011

34. Zaman Q, Khan MA, Sahar K, Rehman G, Khan H, Rehman M, et al. Novel Variants in MPV17, PRX, GJB1, and SACS Cause Charcot-Marie-Tooth and Spastic Ataxia of Charlevoix-Saguenay Type Diseases. Genes (Basel). 2023;14(2).doi:10.3390/genes14020328

35. Nouioua S, Hamadouche T, Funalot B, Bernard R, Bellatache N, Bouderba R, et al. Novel mutations in the PRX and the MTMR2 genes are responsible for unusual Charcot-Marie-Tooth disease phenotypes. Neuromuscul Disord. 2011;21(8):543-50.doi:10.1016/j.nmd.2011.04.013

36. Makrythanasis P, Nelis M, Santoni FA, Guipponi M, Vannier A, Bena F, et al. Diagnostic exome sequencing to elucidate the genetic basis of likely recessive disorders in consanguineous families. Hum Mutat. 2014;35(10):1203-10.doi:10.1002/humu.22617

37. DiVincenzo C, Elzinga CD, Medeiros AC, Karbassi I, Jones JR, Evans MC, et al. The allelic spectrum of Charcot-Marie-Tooth disease in over 17,000 individuals with neuropathy. Mol Genet Genomic Med. 2014;2(6):522-9.doi:10.1002/mgg3.106

38. Schabhuttl M, Wieland T, Senderek J, Baets J, Timmerman V, De Jonghe P, et al. Whole-exome sequencing in patients with inherited neuropathies: outcome and challenges. J Neurol. 2014;261(5):970-82.doi:10.1007/s00415-014-7289-8

39. Boerkoel CF, Takashima H, Stankiewicz P, Garcia CA, Leber SM, Rhee-Morris L, et al. Periaxin mutations cause recessive Dejerine-Sottas neuropathy. Am J Hum Genet. 2001;68(2):325-33.doi:10.1086/318208
